# Clinical outcomes and predictors of success with Impella weaning in cardiogenic shock: a single-center experience

**DOI:** 10.3389/fcvm.2023.1171956

**Published:** 2023-06-21

**Authors:** M. V. Matassini, M. Marini, A. Angelozzi, L. Angelini, M. Shkoza, P. Compagnucci, U. Falanga, I. Battistoni, G. Pongetti, M. Francioni, T. Piva, A. Mucaj, E. Nicolini, A. Maolo, M. Di Eusanio, C. Munch, A. Dello Russo, G. Perna

**Affiliations:** ^1^ Cardiac Intensive Care Unit-Cardiology Division, Cardiovascular Department, Ospedali Riuniti di Ancona, Ancona, Italy; ^2^ Unit of Cardiology and Cardiac Intensive Therapy, Cardiovascular Department, G. Mazzini Hospital, Teramo, Italy; ^3^ Cardiology and Arrhythmology Clinic and Department of Biomedical Sciences and Public Health, University Hospital Ospedali Riuniti di Ancona and Marche Polytechnic University, Ancona, Italy; ^4^ Interventional Cardiology-Cardiology Division, Cardiovascular Department, Ospedali Riuniti di Ancona, Ancona, Italy; ^5^ Cardiac Surgery Unit, Cardiovascular Department, Ospedali Riuniti di Ancona, Ancona, Italy; ^6^ Anaesthesia and Cardiac Surgery Intensive Care, Ospedali Riuniti di Ancona, Ancona, Italy

**Keywords:** heart failure, Impella, weaning, outcomes, complications, cardiogenic shock

## Abstract

**Introduction:**

Cardiogenic shock (CS) is a severe syndrome with poor prognosis. Short-term mechanical circulatory support with Impella devices has emerged as an increasingly therapeutic option, unloading the failing left ventricle (LV) and improving hemodynamic status of affected patients. Impella devices should be used for the shortest time necessary to allow LV recovery because of time-dependent device-related adverse events. The weaning from Impella, however, is mostly performed in the absence of established guidelines, mainly based on the experience of the individual centres.

**Methods:**

The aim of this single center study was to retrospectively evaluate whether a multiparametrical assessment before and during Impella weaning could predict successful weaning. The primary study outcome was death occurring during Impella weaning and secondary endpoints included assessment of in-hospital outcomes.

**Results:**

Of a total of 45 patients (median age, 60 [51–66] years, 73% male) treated with an Impella device, 37 patients underwent impella weaning/removal and 9 patients (20%) died after the weaning. Non-survivors patients after impella weaning more commonly had a previous history of known heart failure (*p* = 0.054) and an implanted ICD-CRT (*p* = 0.01), and were more frequently treated with continuous renal replacement therapy (*p* = 0.02). In univariable logistic regression analysis, lactates variation (%) during the first 12–24 h of weaning, lactate value after 24 h of weaning, left ventricular ejection fraction (LVEF) at the beginning of weaning, and inotropic score after 24 h from weaning beginning were associated with death. Stepwise multivariable logistic regression identified LVEF at the beginning of weaning and lactates variation (%) in the first 12–24 h from weaning beginning as the most accurate predictors of death after weaning. The ROC analysis indicated 80% accuracy (95% confidence interval = 64%–96%) using the two variables in combination to predict death after weaning from Impella.

**Conclusions:**

This single-center experience on Impella weaning in CS showed that two easily accessible parameters as LVEF at the beginning of weaning and lactates variation (%) in the first 12–24 h from weaning begin were the most accurate predictors of death after weaning.

## Introduction

Cardiogenic shock (CS) is a complex and severe clinical syndrome due to a severe impairment of myocardial performance resulting in reduced cardiac output with end-organ hypoperfusion. The goal of CS treatment is to quickly restore cardiac output through a series of historical and established emergency treatments depending on the specific etiology, ranging from volume expansion to vasopressors and inotropes, from early revascularization of the infarct-related artery to intra-aortic balloon pump (IABP) counterpulsation (1–4). In the last decade, the Impella device has emerged as an increasingly therapeutic option for CS (5–12). It is a microaxial, continuous-flow pump, placed across the aortic valve to support and unload the failing left ventricle (LV), with blood flows up to 5.5 L/min. Impella directly unloads the LV, reducing total mechanical work and myocardial oxygen demand, while lowering wall stress and improving subendocardial coronary blood flow (13, 14). These actions favour LV recovery and circulatory stability.

However, mechanical unloading with the Impella device is also complicated by time-dependent device-related adverse events, such as limb ischemia, sepsis, haemolysis, stroke and bleeding. Therefore, the Impella device should be used for the shortest time necessary to allow LV recovery.

The weaning from Impella and its explant, however, are mostly performed in the absence of established algorithms and protocols, mainly based on the experience of the individual centres, and predictors of successful weaning are lacking (15–18).

The aim of this study is to retrospectively evaluate whether a multiparametrical assessment just before and during Impella weaning, including clinical, laboratory, echocardiographic, and hemodynamic data, could predict successful weaning. Furthermore, we aim to describe our experience in the complex field of weaning from Impella, in order to provide guidance in this challenging and largely unexplored critical care scenario.

## Methods

### Patients

The Ancona Impella Registry is a single-center retrospective registry at a high volume tertiary referral hospital with on-site cardiac surgery, including all patients older than 18 years admitted consecutively to the Cardiology Intensive Care Unit (ICU) of the University Hospital “Ospedali Riuniti”, Ancona, from September 2015 to July 2021 because of Cardiogenic Shock, who were supported with an Impella pump (2,5 or CP device; Abiomed Europe GmbH, Aachen, Germany).

The diagnosis of CS was made in the presence of all of the following criteria:

• Systolic blood pressure (SBP) <90 mmHg for ≥30 min OR Support to maintain SBP ≥90 mmHg;
• End-organ hypoperfusion (urine output <30 ml/h, arterial lactate >2 mmol/L, altered mental status or cool extremities);
• Hemodynamic criteria: cardiac index (CI) ≤2.2 L min^−1^ m^−2^ and pulmonary capillary wedge pressure (PCWP) ≥15 mmHg.

The cause of CS was classified as: ischemic (non ST elevation or ST elevation myocardial infarction), related to acute myocarditis or decompensated dilated cardiomyopathy.

### Weaning from Impella

Duration of Impella support was at the discretion of the treating physician, based on the evolving conditions of affected patients, which were re-assessed four times per day or more.

The weaning process was started after hemodynamic stabilization, and in the presence of clinical/instrumental signs of improved cardiac function and end-organ perfusion (5, 8, 17, 19, 20), after a minimum of 48 h of maximal tolerated P-level support.

Weaning was performed by gradually reducing the Impella performance level from P5 to P2. The time when weaning was started was recorded as the onset of weaning.

When P2 level was tolerated for at least 120 min, the device was explanted. The completion of the weaning process usually occurred within 48 h, in absence of new events (as new ischemic clinical events, hypotension with elevate serum lactates and/or metabolic acidosis, reduction in urine output with elevation in serum creatinine, ventricular arrhythmias not related to Impella suction) or failure.

We retrieved baseline demographic variables and medical history, procedural and angiographic information (including, time to balloon, defined as the time between the arrival of a patient with acute coronary syndrome in ICU and the first balloon inflation during percutaneous coronary intervention and time to unload, defined as the time between the arrival of a patient with acute coronary syndrome in ICU and the activation of the impella pump), pharmacological therapy with special attention to inotropes and vasopressor before and during Impella weaning, echocardiographic, laboratory and hemodynamic parameters before and during Impella weaning and in-hospital complications, clinical events and deaths.

We calculated the inotropic score by the standard formula: Dopamine dose (µg/kg/min) + dobutamine dose (µg/kg/min) + 100 × epinephrine dose (µg/kg/min)] + 10 × milrinone dose (µg/kg/min) + 10,000 × vasopressin dose (units/kg/min) + 100 × norepinephrine dose (µg/kg/min).

All data, which were prospectively inserted in our local electronic chart, were therefore included in a pre-specified structured database.

We measured the percent change in serum lactate levels in the first 12–24 h of weaning and named in the results section as “Δ lactate during first 12–24 h of weaning”.

Left ventricular ejection fraction (LVEF) assessed at the onset of weaning was named as “baseline left ventricular ejection fraction”.

All these procedures performed were carried out in accordance with The Code of Ethics of the World Medical Association (Declaration of Helsinki) for experiments involving humans.

### Study endpoints

The primary study outcome was death occurring during Impella weaning.

Secondary endpoints included assessment of in-hospital outcomes (weaning failure, mechanical support escalation, in-hospital deaths and complications).

We defined Impella weaning failure as the need to increase Impella support of at least 1P level because of clinical, hemodynamic and laboratory worsening during Impella support reduction.

Mechanical support escalation was represented by the need to upgrade to a higher-flow support device (veno-arterial extracorporeal membrane oxygenation, ECMO, or left ventricular assist device, LVAD).

In-hospital complications included myocardial re-infarction, arrhythmias, and stroke/transient ischemic attack, access site bleeding, acute limb ischaemia, cardiac tamponade, retroperitoneal hemorrhage or other major bleeding events, clinical significant haemolysis, Impella repositioning, systemic infections and acute kidney injury (AKI).

All bleeding events were classified according to Bleeding Academic Research Consortium (BARC) criteria (21).

Clinical significant haemolysis was defined as the presence of clinical signs (dark urine, scleral icterus, hemodynamic instability) together with laboratory signs of haemolysis (increase of LDH more than 2.5 times compared to baseline value, significant drop in haemoglobin, reduction of haptoglobin, increase of total bilirubin).

### Statistical analyses

Continuous variables were checked for normality using the Shapiro–Wilk test, and are reported as mean ± standard deviation if normally distributed, or as median (1st–3rd quartile) if non-normally distributed.

The association of clinical, echocardiographic, and laboratory parameters with the primary outcome was assessed with univariable logistic regression. Variables associated with primary outcome in univariable analysis with a cut-off *p* value <0.10 were entered into a multivariable model, and retained in the final model according to backward stepwise selection. Performance of the final multivariable logistic regression model was assessed using area under the receiver operating characteristic curve analysis.

Comparisons between groups were performed with the Student *t*-test for normally distributed variables, or Wilcoxon rank sum test for non-normally distributed variables. Youden's index was used to determine the optimal cut-off of quantitative variables for predicting primary outcome events. A 2-sided *p* < 0.05 defined statistical significance. All statistical analyses were performed with the software R (R Foundation for Statistical Computing, Vien, Austria).

## Results

### Patient population

Between September 2015 and July 2021, 45 patients (median age, 60 [51–66] years, 73% male) were treated with an Impella device (Impella CP in 42 patients, 93%, and Impella 2.5 in 3 patients, 7%) for cardiogenic shock, which was already present at hospital admission in 30 cases (67%), or developed during hospitalization in the remaining 15 (33%).

The etiology of CS was mainly ischemic in 37 patients (82%), while acute myocarditis was responsible of 3 cases (7%), and decompensated dilated cardiomyopathy in 5 (11%).

In the setting of acute coronary syndromes, Impella was used as early support before percutaneous coronary intervention (PCI) in 30% of patients.

The main baseline characteristics of registry population are reported in Table 1.

**Table 1:** Baseline characteristics of patients receiving impella support for cardiogenic shock (*n* = 45).

Risk factors and previous medical history	
Age, years, median (Q1–Q3)	60 (51–66)
Male gender, *n* (%)	33 (73)
Diabetes, *n* (%)	10 (22)
Smoking, *n* (%)	23 (51)
Hypertension, *n* (%)	23 (51)
Dyslipidaemia, *n* (%)	20 (44)
Previous acute coronary syndrome, *n* (%)	9 (20)
Previous PCI, *n* (%)	8 (18)
Previous CABG, *n* (%)	1 (2)
Previous heart failure episode, *n* (%)	3 (7)
Previous atrial fibrillation, *n* (%)	4 (9)
PAD, *n* (%)	3 (7)
Previous stroke, *n* (%)	1 (2)
COPD *n* (%)	5 (11)
Clinical and instrumental characteristics on admission	
Acute coronary syndrome, *n* (%)	37 (82)
Myocarditis, *n* (%)	3 (7)
Decompensated dilative cardiomyopathy, *n* (%)	5 (11)
Time from symptoms onset to hospitalization, min, median (Q1–Q3)	300 (110–2,880)
LVEF, %, median (Q1–Q3)	25 (16–30)
TAPSE, mm, median (Q1–Q3)	17 (14–18)
RVFAC, %, median (Q1–Q3)	33 (20–37)
PAPs, mmHg, median (Q1–Q3)	35 (30–43)
Multivessel disease, *n* (%)	24 (53)
PCI as revascularization, *n* (%)	35 (78)
CABG as revascularization, *n* (%)	0 (0)
Lactate value (mmol/L), median (Q1–Q3)	3.7 (2.0–6.2)
Charlson comorbidity index, median (Q1–Q3)	3 (2–5)
Haemoglobin (mg/dl), mean (SD)	12.8 ± 2.3
Troponin (ng/ml), median (Q1–Q3)	4,668 (18–125,000)
Creatinine (mg/dl), median (Q1–Q3)	1.1 (0.9–1.4)
Orotracheal Intubation, *n* (%)	38 (84)
NIV/CPAP, *n* (%)	22 (49)
Inotropic score, median (Q1–Q3)	8 (0–15)
Impella device data	
Impella CP, *n* (%)	42 (93)
Impella 2.5, *n* (%)	3 (7)
Time “door to unloading”, min, median (Q1–Q3)	210 (98–1,118)
Duration of Impella support, h, median (Q1–Q3)	112 (67–192)

The median door to unloading time was 210 min (98–1,118 min), and median time spent with Impella support was 115 (67–200) h.

At admission, the mean Charlson comorbidity index was 4 ± 3, the median LVEF was 25% (15%–60%), median TAPSE 17 mm (10–26 mm), and median RVFAC 33% (15%–40%).

At impella insertion, the mean serum lactate level amounted to 4.4 ± 2.9 mmol/L, mean ScvO2 was 64.8 ± 11.3 mmHg and median inotropic score was 9.5 (0–15).

At the time of device support initiation, the median arterial pressure was 63 (60–70) mmHg, mean heart rate was 108.7 ± 24.3, and 84.3% of patients were on mechanical ventilation.

Inotropic score and serum lactate levels significantly decreased during impella support in total population (inotropic score: 8 [0–15] at baseline, 2 [0–9] after 48 h, *p* = 0.01; serum lactate: 3.7 [2.0–6.2] at baseline, 1.2 [1.0–1.6] after 48 h, *p* = 0.01).

### Weaning from Impella and outcomes

Thirty-seven patients (82%) underwent weaning from Impella or Impella removal during hospitalization.

In fact, because of clinical and/or laboratory worsening, a total of 5 patients (11%) underwent an upgrade to ECMO support, while 2 patients (4.5%) received a durable LVAD. Those cases were considered Impella removal and were not counted in the analysis of Impella weaning.

In the remaining 30 cases, the reasons for weaning were clinical improvement in 22 patients, unmanageable suction alarms in 1 patient, purge pressure alarms in 2 patients, and other complications in 5 patients (1 with major bleeding, 4 with haemolysis).

In 22 of these cases (74%) a single weaning attempt was sufficient, while the rest of patients (*n* = 8, 26%) presented at least an episode of weaning failure and underwent successive attempts, until the device could be safely explanted. The median duration of weaning from Impella was 30 h (0–48).

Seventeen patients (38%) died during hospital stay, and nine patients (20%) died after weaning from Impella.

Considering the deaths after Impella weaning, four deaths were due to refractory cardiogenic shock, one to septic shock, one to refractory ventricular fibrillation, one to acute respiratory distress syndrome and two to intracranial hemorrhage.

The etiology of CS in non-survivors patients after impella weaning was ischemic in 7 cases and decompensated dilated cardiomyopathy in the remaining 2 cases. With regard to ischemic cause, Impella support was implanted before PCI in 3 non-survivor patients.

**Figure 1:**
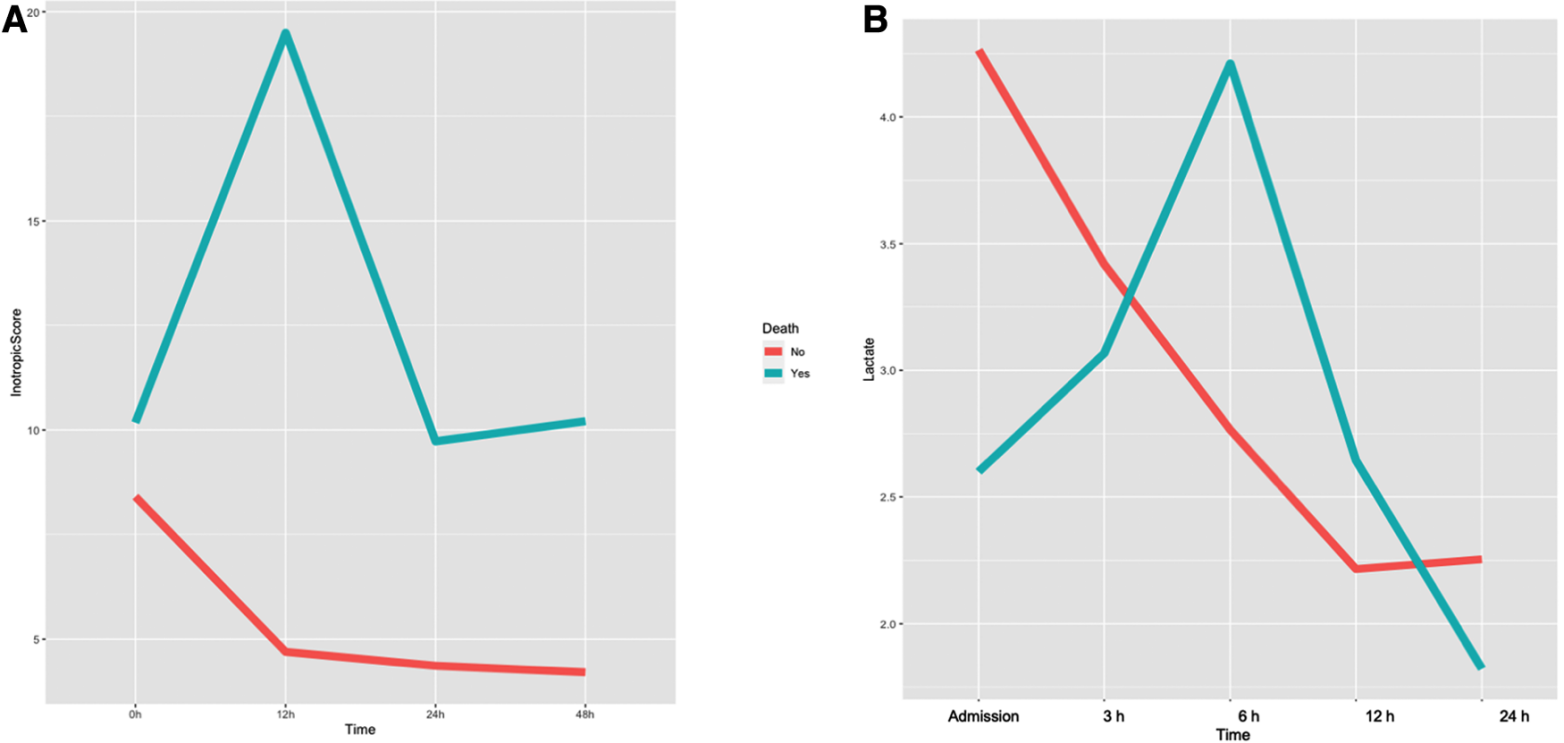
Variation of inotropic score (**A**) and arterial lactates (**B**) during impella support in survivor and non-survivor patients after Impella weaning.

Characteristics of non-survivor patients after Impella weaning when compared to those patients that successfully overcame Impella weaning are reported in Table 2. Inotropic score and serum lactate variations in survivor and non-survivor patients are represented in Figures 1A,B. Non-survivors more commonly had a previous history of known HF (*p* = 0.054) and an implanted ICD-CRT (*p* = 0.01), and were more frequently treated with continuous renal replacement therapy (CRRT; *p* = 0.02).

**Table 2:** Clinical, laboratoristic and instrumental characteristics of non-survivor and survivor patients after Impella weaning.

	Survivors (*n* = 28)	Non-survivors (*n* = 9)	*p*
Age, years, median (Q1–Q3)	57 (44–63)	66 (53–66)	0.10
Male gender, *n* (%)	19 (68)	7 (78)	0.70
Diabetes, *n* (%)	5 (18)	1 (11)	1
Smoking, *n* (%)	15 (54)	4 (44)	0.71
Hypertension, *n* (%)	12 (43)	6 (67)	0.27
Dyslipidaemia, *n* (%)	13 (46)	4 (44)	0.93
COPD, *n* (%)	3 (11)	1 (11)	1
PAD, *n* (%)	1 (4)	0 (0)	1
Ischemic etiology of CS	21 (75)	7 (78)	0.68
ICD/CRT, *n* (%)	0 (0)	3 (33)	**0**.**01**
Previous HF episode, *n* (%)	0 (0)	2 (22)	**0**.**054**
Charlson comorbidity index, mean (SD)	2.9 (1.4)	3.0 (1.4)	0.91
Orotracheal intubation, *n* (%)	22 (79)	8 (89)	0.66
CRRT, *n* (%)	8 (29)	7 (78)	**0**.**02**
NIV/CPAP, *n* (%)	15 (54)	4 (44)	0.71
Pre-PCI Impella implantation	8 (29)	3 (33)	0.22
Duration of Impella support, h, median (Q1–Q3)	120 (74–192)	130 (84–219)	0.55
Positive blood culture, *n* (%)	14 (50)	5 (56)	1
Baseline Haemoglobin (mg/dl), mean (SD))	12.5 (2.2)	13.1 (2.8)	0.58
Baseline Troponin (ng/ml) median (Q1–Q3)	392 (18–103,550)	21,000 (200–200,000)	0.26
Baseline creatinine (mg/dl), mean (SD)	0.98 (0.47)	1.1 (0.7)	0.14
Lactate at impella insertion (mmol/L), mean (SD)	2.8 (2.9)	2.5 (1.3)	0.47
LVEF (%) at Impella insertion, mean (SD)	21.5 (6.3)	20 (10)	0.13
TAPSE (mm) at Impella insertion, mean (SD)	16.4 (3.5)	16.1 (3.8)	0.83
RVFAC (%) at Impella insertion, mean (SD)	34 (13)	31 (10)	0.70
Inotropic score at Impella insertion, mean (SD),	4 (14)	11 (5)	0.35
PAPs (mmHg) median (Q1–Q3)	35 (30–40)	38 (31–45)	0.71

Bold values represents statistically significant *p* values.

In univariable logistic regression analysis, Δ lactate during the first 12–24 h of weaning, lactate value after 24 h of weaning (per unit variation), baseline LVEF (per unit variation), and inotropic score after 24 h of weaning (per unit variation), were associated with death, as reported in Table 3.

**Table 3:** Univariable predictors of death after weaning, selected for having univariable *p* < 0.10.

Variable	OR	Lower CL	Upper CL	*p* Value
Δ lactate during first 12–24 h of weaning (per 100% variation)	10.84	1.17	100.80	0.036
Lactate after 24 h of weaning (per unit variation)	6.32	1.02	39.30	0.048
LVEF at the onset of weaning (per unit variation)	0.88	0.77	1.00	0.056
Inotropic score after 24 h of weaning (per unit variation)	1.07	0.99	1.15	0.082
Time quartile of hospital admission	0.897	0.795	1.011	0.086

Stepwise multivariable logistic regression identified baseline LVEF and Δ lactate during the first 12–24 h of weaning as the most accurate predictors of death after weaning (Table 4).

**Table 4:** Multivariable predictors of death after weaning.

Variable	OR	Lower CL	Upper CL	*p* Value
LVEF at the onset of weaning (per unit variation)	0.87	0.76	0.99	0.039
Δ lactate during first 12–24 h of weaning (per 100% variation)	25.11	1.2	524.32	0.038

**Figure 2:**
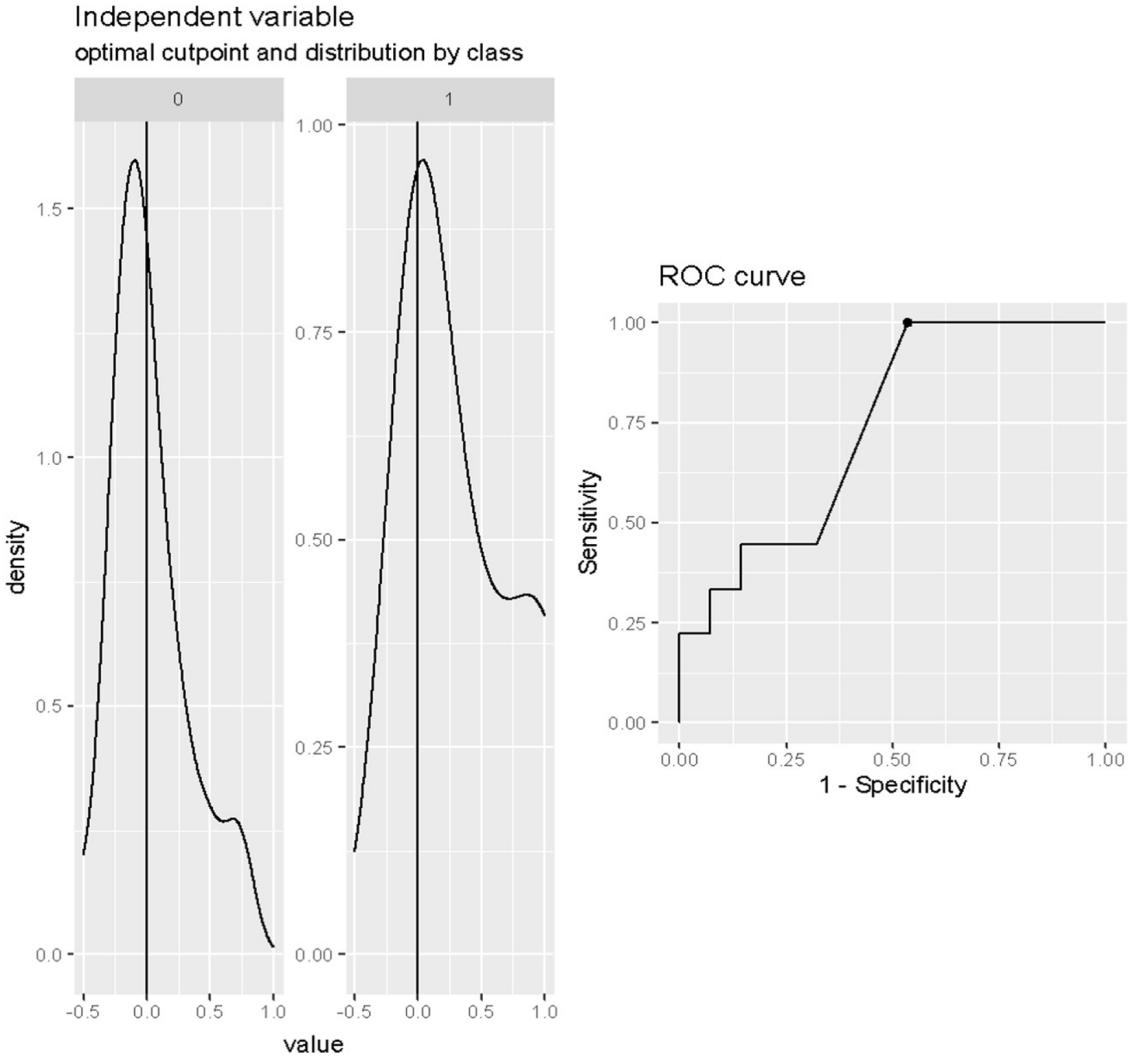
Cut-off analysis of Δ lactate (%) during first 12–24 h of weaning in predicting death after weaning. Cut-off = 0%; SE = 1; SP = 0.46; accuracy = 0.5946.

The optimal cut-off value of the Δ lactate during the first 12–24 h of weaning for the prediction of death after weaning was any value more than 0% (Cut-off = 0%; SE = 1; SP = 0.46; accuracy = 0.5946), as shown in Figure 2.

**Figure 3:**
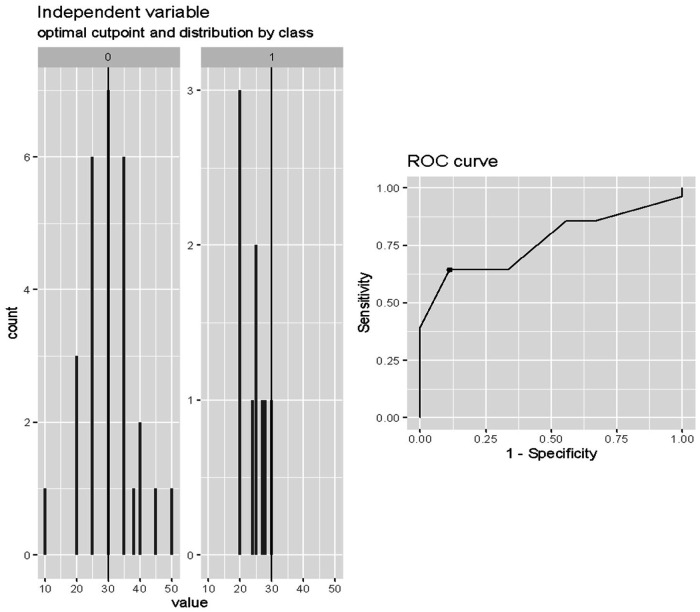
Cut-off analysis of LVEF at onset of weaning in predicting death after weaning. Cut-off = 0.30; SE = 0.64; SP = 0.89; accuracy = 0.70.

The optimal cut-off value of the LVEF in predicting death after weaning was 30% (Cut-off = 0.30; SE = 0.64; SP = 0.89; accuracy = 0.70) as reported in Figure 3.

**Figure 4:**
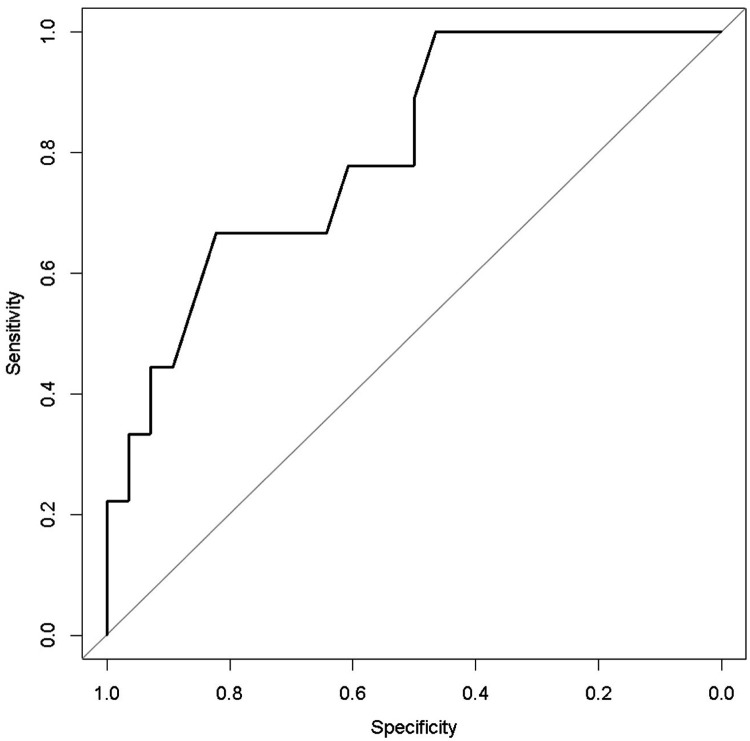
Receiver operating characteristic curve analysis of the multivariable prediction model for death after weaning from impella (accuracy = 0.80).

The ROC analysis indicated 80% accuracy (95% confidence interval = 64%–96%) using the two variables in combination to predict death after weaning from Impella (Figure 4).

### Patients' characteristics with unsuccessful first attempt of Impella weaning

As reported in Table 5, patients with an unsuccessful first attempt of Impella weaning, despite similar baseline characteristics (age, gender, comorbidities) when compared to patients who positively achieved a first attempt of weaning, presented a longer total duration of Impella support (*p* = 0.006) and weaning (*p* = 0.0098), higher level of maximum creatinine (*p* = 0.0167), higher lactate at impella insertion (*p* = 0.067), and inotropic score at onset of weaning (*p* = 0.047).

**Table 5:** Characteristics of patients with unsuccessful and successful first attempt of Impella weaning.

	Successful first attempt of Impella weaning (*n* = 22)	Unsuccessful first attempt of Impella weaning (*n* = 8)	*p*
Age, years—mean (SD)	58 (48–65)	60 (57–67)	0.76
Female gender, *n* (%)	5 (23)	3 (38)	0.64
Diabetes, *n* (%)	4 (18)	2 (25)	0.65
Smoking (past or present), *n* (%)	13 (59)	5 (63)	1
Hypertension, *n* (%)	10 (46)	5 (63)	0.68
Dyslipidaemia, *n* (%)	12 (55)	3 (38)	0.68
COPD, *n* (%)	3 (14)	1 (13)	1
PAD, *n* (%)	1 (5)	0 (0)	1
ICD/CRT, *n* (%)	1 (5)	0 (0)	1
Previous HF episode, *n* (%)	0 (0)	0 (0)	1
Charlson comorbidity index. mean (SD)	3.0 (1.9)	3.4 (1.2)	0.53
Orotracheal intubation, *n* (%)	17 (77)	7 (88)	0.92
CRRT, *n* (%)	6 (27)	4 (50)	0.38
NIV/CPAP, *n* (%)	16 (73)	6 (75)	1
Total Duration of Impella support, h, median (Q1–Q3)	94 (69–146)	201 (179–227)	**0**.**006**
Hours with the highest Impella P level, median (Q1–Q3)	12 (6–29)	39 (20–53)	0.15
Total duration of weaning, h, mean (SD)	34.8 (20.9)	76.4 (33.8)	**0**.**0098**
Positive blood culture, *n* (%)	10 (46)	4 (50)	1
Baseline Haemoglobin (mg/dl), mean (SD)	13.2 (2.3)	12.7 (2.1)	0.56
Baseline Troponin (ng/ml), median (Q1–Q3)	11,417 (82–148,250)	10,600 (50–125,000)	0.62
Troponin at onset of weaning, median (Q1–Q3)	7,254 (19–36,765)	57 (2–15,304)	0.32
Baseline creatinine (mg/dl), median (Q1–Q3)	0.98 (0.78–1.23)	1.2 (1.04–1.44)	0.17
Maximum creatinine (mg/dl), median (Q1–Q3)	1.32 (0.94–3.03)	4.13 (1.73–4.92)	**0**.**0167**
Baseline Lactate, Median (Q1–Q3)	2.1 (1.4–4.9)	3.3 (2.3–5.1)	0.17
Lactate at impella insertion, median (Q1–Q3)	2.4 (1.8–3.4)	4.1 (2.5–5.1)	**0**.**067**
Lactate at onset of weaning, median (Q1–Q3)	1.0 (0.8–1.3)	1.2 (0.9–1.2)	0.60
Heart rate at onset of weaning, mean (SD)	87 (16)	88 (18)	0.96
Mean arterial pressure at onset of weaning, median (Q1–Q3)	71 (65–75)	73 (72–75)	0.45
LVEF (%) at Impella insertion, median (Q1–Q3)	22 (20–29)	20 (15–25)	0.38
LVEF (%) at onset of weaning, median (Q1–Q3)	30 (25–35)	26 (24–30)	0.20
TAPSE (mm) at Impella insertion, mean (SD)	17 (3)	17 (3)	0.79
TAPSE (mm) at onset of weaning, median (Q1–Q3)	19 (17–19)	19 (17–19)	0.72
RV dysfunction during Impella support, *n* (%)	7 (32)	2 (25)	1
Inotropic score at Impella insertion, median (Q1–Q3)	4 (0–10)	10 (4–16)	0.25
Inotropic score at onset of weaning, median (Q1–Q3)	3 (0–10)	14 (9–18)	**0**.**047**
PAPs at Impella insertion (mmHg), median (Q1–Q3)	40 (33–40)	30 (30–36)	0.29

Bold values represents statistically significant *p* values.

### In-hospital complications

No major device malfunctions were reported in the entire population. Displacement of Impella requiring repositioning procedures occurred in 23 cases.

Red blood cell transfusion was the most frequent event for the entire cohort (70%). Serial assessment of haptoglobin levels revealed an overall incidence of haemolysis in 51% of patients, although clinically significant haemolysis occurred in 4 cases (8.8%).

No retroperitoneal hemorrhage (RPH) was reported, while a patient (2.2%) developed cardiac tamponade during Impella support. Bleeding at the Impella access site was described in 14 patients (31%), and six patients (13%) experienced acute limb ischaemia.

Two patients were diagnosed with ischemic stroke (4.4%) and other 2 with haemorrhagic stroke (4.4%).

Continuous renal replacement therapy (CRRT) was required in 16 patients (36%).

Twenty-eight patients (62%) developed fever during Impella support but only 12 of them (42%) presented positive blood cultures.

### In hospital mortality trend analysis

**Figure 5:**
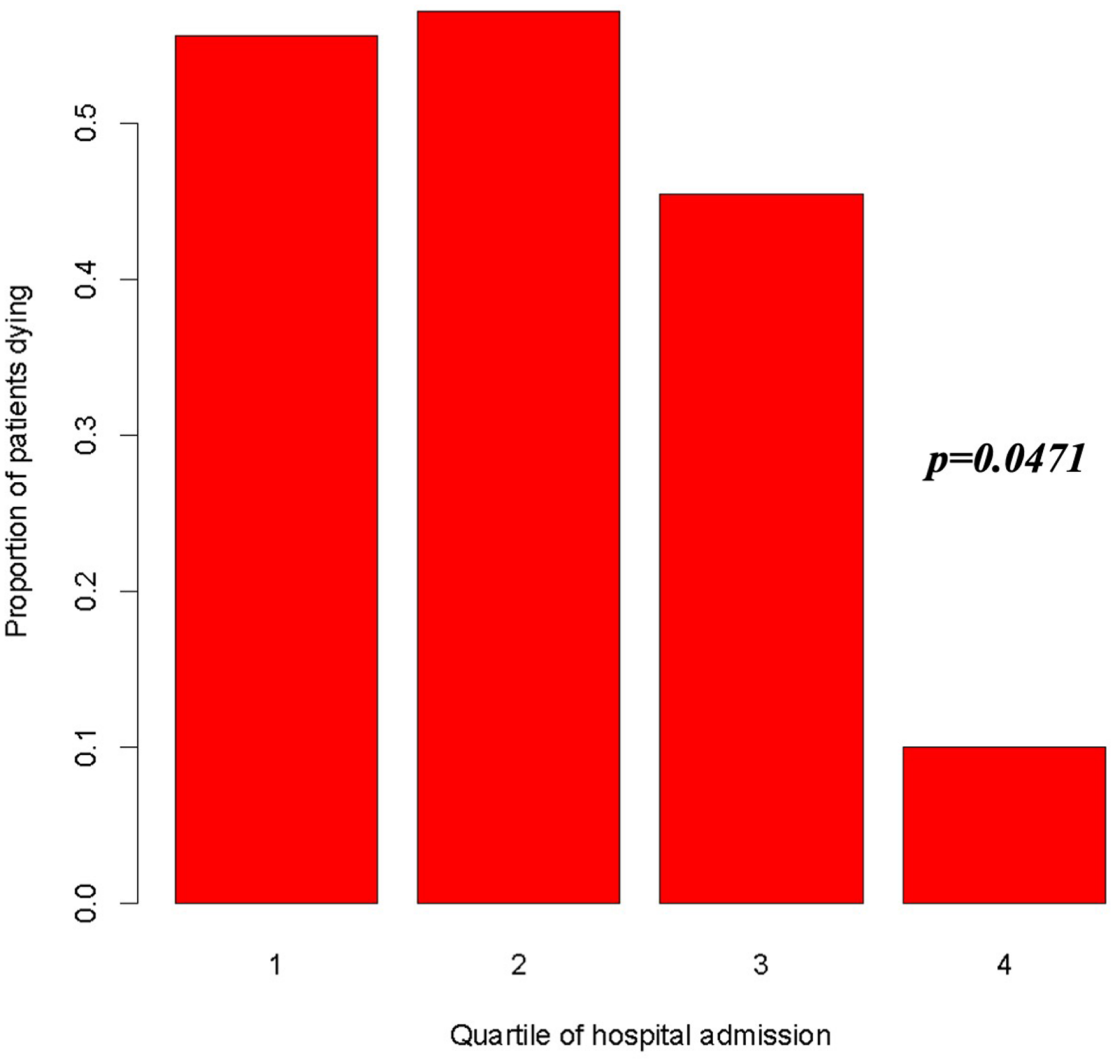
Risk of death according to quartile of admission period (1st quartile: April 28, 2014–August 20, 2018; 2nd quartile: August 20, 2018–October 14, 2019; 3rd quartile: October 14, 2019–July 12, 2020; 4th quartile: July 12, 2020–July 3, 2021). OR = 0.523, 95%, CI = 0.275–0.992, *p* = 0.0471.

A clear temporal trend in in-hospital mortality was evident when considering the rate along time defined as quartile (April 2014–August 2018; August 2018–October 2019; October 2019–July 2020; July 2020–July 2021), with a significant reduction in the risk of death as reported in Figure 5 (OR = 0.523, 95%, CI = 0.275–0.992, *p* < 0.05).

## Discussion

In this retrospective study we found that two easily accessible parameters could accurately predict the risk of death after weaning.

As Impella use is rapidly increasing among patients with CS, it is urgent to define the right way to perform the weaning and predict a successful process until explantation. Clinical judgment is not enough to accurately predict patient outcomes.

The weaning criteria differ widely among centres. A recent survey reported that surrogates of hemodynamic stability and end-organ perfusion are the most commonly used parameters to guide the weaning process, which is usually considered in the presence of adequate oxygenation and ventilation, followed by the lowest need of vasoactive agent (18). However, the same authors underlined the numerous knowledge gaps in this field, especially the paucity of data correlating hemodynamic estimates to imaging variables of ventricular recovery and, most importantly, to clinical outcomes.

In this scenario, our study revealed that an imaging criterion (LVEF at onset of weaning) and an organ perfusion surrogate (Δ lactate during the first 12–24 h of weaning) were the most accurate predictors of death after weaning with an accuracy of 80% when the two variables were taken together. Both parameters are easily accessible and their use could help every cardiologist dealing with cardiogenic shock and Impella support.

Any increase in lactates should trigger a prompt answer, postponing the weaning process or modifying drugs therapy while reducing circulatory support. No studies have previously defined the entity of arterial lactate increase during weaning correlating with death: we found that any increase is associated with an unsuccessful weaning process.

In a similar manner, the LVEF cut-off value correlating with fatal outcome was 30%.

The persistence of a severe left ventricular systolic dysfunction during Impella unloading is the expression of a pronounced and serious alteration of pump function and should push cardiologists to carry out alternative strategies to prevent the failure of weaning as a inodilator infusion [for instance levosimendan, as it commonly happens in venoarterial ECMO weaning (22–24)] or, in the worst cases the upgrade to an ECMO support or consideration for long-term LV support or heart transplantation, after a case-by-case discussion.

We also found that patients with first unsuccessful attempt of Impella weaning presented a longer duration of Impella support and weaning, worst metabolic and organ characteristics and higher gravity in term of inotropic score, underscoring the greater general complexity of these patients.

Our registry also provides insight into complications of patients treated with Impella.

We describe an overall in-hospital mortality of 38%, that is lower when compared with other registry data (10, 25, 26). Moreover we found a significant reduction in the risk of death over time.

Complications rate is in agreement with previous reports (5–10) and consisted of bleeding at the Impella access site (31%), acute limb ischaemia (3%), clinical significant haemolysis (8.8%), stroke (8.8%) and cardiac tamponade (2.2%). More than half of patients (62%) developed fever during Impella support but 42% of them presented positive blood cultures.

Management and monitoring of such devices requires a level of long-term expertise in high volume tertiary centres with 24 h/7 days availability of trained intensivists and echocardiographers, together with cardiac and vascular surgeons to manage complications.

## Limitations

There are some important limitations to consider. First, the nature of reported data is observational from a retrospective registry, and causal relation between Impella weaning and outcomes cannot be ascertained. Moreover, our study involved a small number of patients in a single center, and all therapeutic decisions were left to the treating physicians' discretion, in the absence of a standardized protocol; all these aspects could arise the possibility of selection bias. However, patient management was in line with expert consensus recommendations, in a field in which no patient-level data is currently available. Our data may represent an important preliminary experience in the complex field of weaning from mechanical circulatory support, and stimulate further clinical research.

## Conclusions

This single-center experience on Impella weaning in cardiogenic shock showed that two easily accessible parameters as LVEF at onset of weaning and a change in serum lactate levels during the first 12–24 h of weaning were the most accurate predictors of death after weaning. In the absence of a defined and universally recognized weaning protocol, the use of these two widely available parameters could help in the identification of the appropriate timing and performance of Impella weaning.

## Data Availability

The raw data supporting the conclusions of this article will be made available by the authors, without undue reservation.
